# "A novel *in vivo *model for the study of human breast cancer metastasis using primary breast tumor-initiating cells from patient biopsies"

**DOI:** 10.1186/1471-2407-12-10

**Published:** 2012-01-10

**Authors:** Carolyn G Marsden, Mary Jo Wright, Latonya Carrier, Krzysztof Moroz, Radhika Pochampally, Brian G Rowan

**Affiliations:** 1 Department of Structural and Cellular Biology, Tulane University Health Sciences Center, The Louisiana Cancer Research Consortium, New Orleans, LA 70112, USA; 2 Department of Surgery, Tulane University School of Medicine, The Louisiana Cancer Research Consortium, New Orleans, LA 70112, USA; 3 Department of Structural and Cellular Biology, Tulane University Health Sciences Center, New Orleans, LA 70112, USA; 4 Section of Surgical Pathology & Cytopathology, Tulane University School of Medicine, Louisiana Cancer Research Consortium, New Orleans, LA 70112, USA; 5 Department of Pharmacology, Tulane University Health Sciences Center, Center for Gene Therapy, Louisiana Cancer Research Consortium, New Orleans, LA, 70,112, USA; 6 Department of Structural and Cellular Biology, Tulane University Health Sciences Center, Louisiana Cancer Research Consortium, Center for Gene Therapy, New Orleans, LA 70112, USA

**Keywords:** Primary breast tumor-initiating cells, Metastasis, Dormancy, EMT

## Abstract

**Background:**

The study of breast cancer metastasis depends on the use of established breast cancer cell lines that do not accurately represent the heterogeneity and complexity of human breast tumors. A tumor model was developed using primary breast tumor-initiating cells isolated from patient core biopsies that would more accurately reflect human breast cancer metastasis.

**Methods:**

Tumorspheres were isolated under serum-free culture conditions from core biopsies collected from five patients with clinical diagnosis of invasive ductal carcinoma (IDC). Isolated tumorspheres were transplanted into the mammary fat pad of NUDE mice to establish tumorigenicity *in vivo*. Tumors and metastatic lesions were analyzed by hematoxylin and eosin (H+E) staining and immunohistochemistry (IHC).

**Results:**

Tumorspheres were successfully isolated from all patient core biopsies, independent of the estrogen receptor α (ERα)/progesterone receptor (PR)/Her2/neu status or tumor grade. Each tumorsphere was estimated to contain 50-100 cells. Transplantation of 50 tumorspheres (1-5 × 10^3 ^cells) in combination with Matrigel into the mammary fat pad of NUDE mice resulted in small, palpable tumors that were sustained up to 12 months post-injection. Tumors were serially transplanted three times by re-isolation of tumorspheres from the tumors and injection into the mammary fat pad of NUDE mice. At 3 months post-injection, micrometastases to the lung, liver, kidneys, brain and femur were detected by measuring content of human chromosome 17. Visible macrometastases were detected in the lung, liver and kidneys by 6 months post-injection. Primary tumors variably expressed cytokeratins, Her2/neu, cytoplasmic E-cadherin, nuclear β catenin and fibronectin but were negative for ERα and vimentin. In lung and liver metastases, variable redistribution of E-cadherin and β catenin to the membrane of tumor cells was observed. ERα was re-expressed in lung metastatic cells in two of five samples.

**Conclusions:**

Tumorspheres isolated under defined culture conditions from patient core biopsies were tumorigenic when transplanted into the mammary fat pad of NUDE mice, and metastasized to multiple mouse organs. Micrometastases in mouse organs demonstrated a dormancy period prior to outgrowth of macrometastases. The development of macrometastases with organ-specific phenotypic distinctions provides a superior model for the investigation of organ-specific effects on metastatic cancer cell survival and growth.

## Background

Breast cancer is a heterogeneous disease that remains the second leading cause of death among women. Metastatic disease increases mortality from breast cancer by 70% and is the leading cause of death in breast cancer patients independent of the manageability of the primary disease. Although generally correlated with later stages in disease progression, there is mounting evidence suggesting the metastatic process may initiate earlier in breast cancer development. Therefore, tumor volume at diagnosis may not accurately predict the presence of metastatic disease or the initiation of the metastatic process. Metastatic disease can remain dormant and undetectable for months to years, resulting in recurrence at the primary site and/or the development of metastatic lesions at distant sites [1,2]. Efficacious treatments for metastatic disease depends on development of preclinical tumor models that better predict patient response, increase understanding of the metastatic process, and enable the identification of biomarkers for earlier and more accurate detection of metastasis.

The study of breast cancer has depended heavily upon the use of established breast cancer cell lines, whose origin is often from pleural effusions or metastatic lesions. Although significant advancements have been made possible through the use of established cell lines, further progress depends on the development of tumor models that more accurately represent the heterogeneous nature of human breast tumors. Hetero-transplantation of primary tumor biopsies from patients into immune-deficient mice has many advantages over standard xenografts from cancer cell lines. The hetero-transplant tumors can be directly compared to the original patient tumor biopsies, and to annotated information on patient features, family history, patient outcome etc. A study of breast cancer hetero-transplants revealed that patients whose breast cancer biopsies grew as tumors in mice predicted a worse prognosis compared to biopsies that did not grow tumors [3]. Unfortunately, only a very small percentage of human breast tumor tissue directly transplanted into immune-deficient mice results in tumor formation [3-5]. The identification of breast cancer stem cells (bCSCs) in breast tumors shifted the previously held hypothesis that all cells within a tumor retained the ability to recapitulate the tumor [6]. BCSCs, present in tumors at very low frequency [7], have been implicated in breast tumor progression [8], metastasis [9] and recurrence [10]. The relative quiescence of bCSCs [11] and the elevated expression of ABC transporter family of proteins [12] may contribute to bCSCs evasion of traditional chemotherapy and radiotherapy. Furthermore, recent data has shown that chemotherapeutics [13,14] and radiation [15] may enrich for bCSCs, possibly increasing risk of recurrence.

A subset of cells isolated from primary breast tumors are termed breast tumor-initiating cells (bTICs) for the ability to form tumors upon injection of low numbers into the mammary fat pad of immune-deficient mice [7]. BTICs consist of a heterogeneous population of cells that include a small percentage of bCSCs as well as a range of less to more differentiated progenitor cells. BTICs have been shown to exist *in vitro *as tumorspheres upon selection under non-adherent, serum-free conditions [16]. Recently, it has been suggested that bTICs are the cells within tumors with metastatic potential and the ability to "seed" in distant organs [17]. Therefore, the challenges in targeting bTICs likely extend from the primary site of tumor formation to distant metastatic sites as well. Given the evidence that supports bTICs as the cells with metastatic potential and the source of breast cancer recurrence, tumor models that employ bTICs isolated directly from patient biopsies may provide a more reliable means for study of the metastatic process and tumor recurrence.

Disseminated breast cancer cells may be present at distant sites at the time of primary diagnosis of breast cancer in patients that exhibit no outward signs of clinical metastasis [18,19]. Although current models of breast cancer metastasis have provided great insight into some of the contributing molecular mechanisms, these models have failed to recapitulate the dormancy period observed clinically. Exit from the dormant state is necessary for the development of macro-metastatic lesions in distant organs, yet the mechanisms involved are poorly understood [17,20,21]. The purpose of this study was to develop a novel and reproducible breast cancer model using bTICs isolated as tumorspheres from patient biopsies for the investigation of the metastatic process.

## Methods

### Isolation of tumorspheres

Tumorspheres were isolated using a procedure previously described by this laboratory [22] and derived from Dontu et al. [23]. Briefly, breast cancer needle biopsies from primary patient tumors were obtained during the routine care of patients with consent and Tulane IRB approved protocol (IRB # 07-00042). Biopsies were performed using a 14 gauge spring-loaded gun yielding about 15-20 mg of tissue in each core sample. 3-5 core biopsy samples (2 cm in length) were obtained from each consenting patient. Tissue samples were placed on ice in 1× Hanks buffered saline solution (HBSS) until processing. Tissues were mechanically dissociated using sterile scalpels into ~2 mm^2 ^pieces followed by enzymatic dissociation in collagenase (300 U/ml) and hyaluronidase (100 U/ml) (Stem Cell Technologies) diluted in complete DMEM/F12 media (see below) for 3-5 h at 37 deg with agitation every 20-25 min. The resultant cell suspension was sequentially filtered through a 100 μm and 40 μm pore filter (Fisher) and centrifuged at 300 × g for 10 min. The cell pellet was resuspended in complete DMEM/F12 media (see below) and cultured in a 100 mm^2 ^ultra low attachment plate (Corning).

### Cell culture

Cells isolated from tissue samples were incubated in DMEM/F12 media containing 1× B-27 serum-free supplement (Invitrogen), 0.4% bovine serum albumin (BSA) (Sigma), 20 ng/ml epidermal growth factor (EGF) (Sigma), 10 ng/ml basic fibroblast growth factor (bFGF) (Sigma), 4 ug/ml insulin, human recombinant (Sigma), and Penicillin (100 U/ml)/Streptomycin (100 U/ml). Cells were cultured for 10-14 days to allow tumorsphere formation. Cells were pelleted every 3 days by centrifugation at 300 × g for 10 min. and resuspended in complete DMEM/F12 media supplemented with fresh EGF and bFGF.

### Animal experiments

Immunodeficient Nu/Nu female mice were purchased from Charles River Laboratories (US). Mice were 25-35 days of age at time of tumorsphere injection. All experiments were performed under approved Tulane IACUC protocol (IACUC # 2941 R-D). Tumorspheres were washed twice with cold 1× PBS then resuspended to yield 1000-5000 cells/100 μl in cold 1× PBS. Immediately before injection, cells were combined with 100 μl BD Matrigel Basement Membrane matrix (BD Biosciences). Mice were anesthetized by i.p. injection of 0.3 ml of a ketamine solution. Cell suspensions were injected bilaterally into the third mammary fat pad. Mice were monitored weekly for tumor formation by caliper measurement, and for body weight for up to 12 months. If no weight loss or other indications of declining health was observed, animals were euthanized 12 months post injection to permit detection of metastases, and to harvest fresh tumor for serial transplantation.

### DNA isolation and PCR analysis of tumors and mouse tissues

For DNA analysis, tissues were collected using autoclaved dissection tools and placed immediately into sterile polypropylene 15 ml conical tubes at a ratio of 5 ml RNAlater (Qiagen) per 200 mg of tissue. Tissues were either processed immediately or stored at -20 deg. Tissues were homogenized using an electric homogenizer (TH-01, Omni TH, tissue homogenizer) at 25,000 rpm for 2 min. with autoclaved Omni homogenizer tips (8 mm diameter, 110 mm length, processing range of 0.25-30 ml) (Omni International). Cell lysis and all subsequent steps for the isolation of DNA and RNA from the homogenized tissues were carried out as described in the instruction manual for the Allprep DNA/RNA isolation kit (Qiagen). Briefly, homogenized tissues were loaded onto a spin column that bound DNA and eluted the fraction containing RNA following centrifugation at room temperature. The eluent was combined with 70% ethanol and added to a 2nd spin column. Following several washes, RNA was eluted and quantified. DNA was also eluted from the 1st spin column and quantified. Human cells were detected in mouse tissues using PCR for detection of an alpha-satellite DNA sequence of the centromere region of human chromosome 17 as previously described by Becker et al [24].

### Hematoxylin and eosin staining

Tissues were collected and placed in 10% neutral buffered formalin (Fisher) equal to 20 times the tissue volume. Tissues were incubated overnight at room temperature and then processed by standard formalin fixation, paraffin embedding and sectioning by The Center for Gene Therapy Histology Core Facility, Tulane University. 5 μm sections were deparaffinized and rehydrated in a graded series of ethanol solutions, from 100% to 75%. Sections were then stained using Gill's Hematoxylin and Eosin (Poly Scientific) followed by dehydration through a graded series of ethanol solutions from 75% to 100%. Image J software was used to quantify the metastatic burden within the tissues analyzed. To calculate the metastatic burden present in the mouse organs, the number of pixels within the defined area of the metastatic lesion/s was determined (x pixels). Next, the total number of pixels within the field of view was determined (y pixels). The metastatic burden within the field of view was then calculated by dividing the pixels present in the metastatic lesion/s, by the total pixels comprising the field of view then multiplying by 100 [(x pixels/y pixels)*100] resulting in a percent metastatic burden. The average of 5 fields of view (100× magnification) was used to determine metastatic burden present in each organ analyzed.

### TUNEL (TdT-mediated dUTP nick end labeling)

For TUNEL, 5 μm sections were rehydrated (as described above) followed by heat-induced, epitope retrieval performed in a pressure cooker for 45 min in pH 6.0 Citrate buffer (Biocare Medical). Sections were allowed to cool for 20 min at room temperature. Sections were immersed for 30 min at room temperature in blocking solution in 0.1 M Tris-HCl (pH 7.5) containing 3% BSA and 20% fetal bovine serum (FBS). Sections were rinsed twice with PBS at room temperature. Positive control sections were incubated with 0.5 mg/ml DNase I diluted in a buffer containing 10 mM Tris-HCl (pH 7.5), 1 mM MgCl_2 _and 1 mg/ml BSA at room temperature for 10 min. The sections were incubated in the TUNEL reaction mixture (as supplied by *In Situ *Cell Death Detection kit, Roche) for 60 min at 37°C in a humidified chamber. Sections were washed three times for 5 min in PBS, and then incubated for 10 min at room temperature in 0.3% H_2_O_2 _diluted in methanol. To block nonspecific binding of the anti-fluorescein antibody, the sections were incubated in blocking solution (as described above) for 30 min at room temperature then rinsed in PBS. Sections were incubated in Converter-POD (as supplied by *In Situ *Cell Death Detection kit, Roche), diluted in blocking solution (as described above), for 30 min at 37°C in a humidified chamber. Sections were rinsed three times for 5 min each in PBS. The signal was developed using the Vector DAB substrate kit, according to the manufacturers' instructions. Sections were dehydrated (as described above) and mounted using Permount (Fisher).

### Immunohistochemistry of tumors and mouse tissue

For immunohistochemistry (IHC), tissues were collected and placed in 10% neutral buffered formalin equal to 20 times the tissue volume (Fisher). Tissues were incubated overnight at room temperature and then processed by standard formalin fixation, paraffin embedding and sectioning by The Center for Gene Therapy Histology Core Facility, Tulane University. Immunohistochemistry was performed as per the Vectastain staining kit (anti-rabbit, PK6101; anti-mouse PK6102, Vector Laboratories). Briefly, 5 μm sections were rehydrated (as described above) followed by heat-induced, epitope retrieval performed in a pressure cooker for 25 min. in Tris Buffer, pH 9 (Biocare Medical) or 45 min in pH 6.0 Citrate buffer (Biocare Medical). To inactivate the endogenous peroxide, slides were incubated in 0.3% hydrogen peroxide followed by a 10 min wash in dH_2_O then washed 3 × 3 min. each in PBS. Sections were incubated in blocking buffer (10% normal goat serum diluted in PBS) for 30 min. at room temp and subsequently incubated with primary antibody diluted in blocking buffer overnight at 4 deg. Primary monoclonal antibodies used were rabbit anti-human Ki-67 (SP6, Thermo Scientific), rabbit anti-human E-cadherin (24E10, Cell Signaling), rabbit anti-human Vimentin (SP20, Vector Laboratories), rabbit anti-human Estrogen receptor (SP1, Thermo Scientific), and anti-human HNA (MAB1281, Chemicon). Primary polyclonal antibodies used were rabbit anti-human β-catenin (9563, Cell Signaling), rabbit anti-human fibronectin (ab2413, Abcam), and rabbit anti-human (human specific) cytokeratin 8 (ab52949, Abcam). The following day, sections were washed 2 × 5 min. in PBS-T. Biotinylated secondary antibody was added to the sections for an incubation period of 30 min, followed by 2 × 5 min. washes in PBS-T. Streptavidin/biotin HRP-conjugate was added to the sections for an incubation period of 30 min. at room temperature followed by 2 × 5 min. washes in PBS-T. The signal was developed using the Vector DAB substrate kit, according to the manufacturers' instructions. Sections were dehydrated (as described above) and mounted using Permount (Fisher). Staining was visualized using a bright field microscope and IP lab software. For quantitation, five randomly selected bright field microscope images (magnification 200×) per sample were obtained as described above. The total cell number in each image was calculated by counting hematoxylin-positive cells using Image J particle count command, and DAB-positive cells were also counted the same way after performing color deconvolution command and expressed as % positive cells.

### Histological scoring

Her 2 expression within the tumors was measured by IHC and assessed using the histoscore method developed by Allred *et. al. *[25]. Briefly, a proportion score and an intensity score were determined for each tumor sample. The proportion score represented the percentage of positively stained cells (0 = none; 1 = < 5%; 2 = 5-25%; 3 = 26-50% 4 = 51-75% 5 = > 75%) [26]. The intensity score represented the staining intensity in positively stained cells (0 = none; 1 = + weak; 2 = ++ intermediate; 3 = +++ strong). The overall expression of Her2 in each tumor sample was reported as a histoscore, calculated by the sum of the proportion score (0-5) and the intensity score (0-3) for a range between 0 and 8, with a maximum possible score of 8 [25].

## Results

### Isolation of tumorspheres from human breast core biopsies

**Figure 1:**
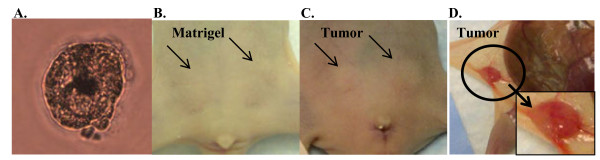
**Tumor formation in the mammary fat pad upon injection of human tumorspheres**. **A**. Light microscopy of a representative tumorsphere isolated from a patient core biopsy following *in vitro *culture for 10 days. **B**. Injection of Matrigel alone into the 3rd mammary fat pad of female NUDE mice. **C, D**. ≤5 × 10^3 ^cells derived from the original patient biopsy were injected into the 3rd mammary fat pad in the form of 'tumorspheres' (with Matrigel) and resulted in formation of small, palpable tumors within 3 months injection with an approximate, sustainable tumor volume of 100 mm^3^

The purpose of this study was to establish a reproducible method for the isolation of primary tumorspheres from patient core biopsies and characterization of subsequent tumor formation upon transplantation into nude mice. The establishment of a hetero-transplantation model, as described in this report, provides an improved and translatable murine model for the study of human breast cancer metastasis. Patient core biopsies were obtained using a 14-gauge spring-loaded gun, yielding 15-20 mg of tissue per biopsy. Tumorspheres were derived from patient core biopsies under serum-free, non-adherent culture conditions as described in Materials and Methods. The ER/PR/Her2 status differed in the patient samples (Table 1). The majority of patient samples were Grade 2 or higher and diagnosed as invasive ductal carcinoma (IDC) (Table 1). Tumorspheres were successfully isolated from all patient samples and ranged in size from 30 μm to 100 μm (Figure 1A). The isolated tumorspheres demonstrated a cell surface marker phenotype CD44^+^/CD24^med/low^/ESA^+ ^by immunocytochemistry (Additional file 1: Figure S1a-b), a phenotype previously determined to be tumorigenic in immune-deficient mice [27].

**Table 1:** Formation of primary tumor and metastasis in NUDE mice implanted with tumorspheres isolated from human breast core biopsies.

Sample	Formation in mice	Latency to palpable tumor formation	Passage in Mice	Metastasis	Metastatic Latency	Age	Diagnosis	Grade	ER/PR/Her2 Status
4	0/2	N/A	N/A	Not Determined	N/A	56 years	IDC	Grade 2	ER^+^/PR^+^/Her2^-^
5	5/6	74 days	Yes	Yes	254 days	44 years	IDC	Grade 3	ER^+^/PR^+^/Her2^-^
6	6/6	47 days	Yes	Yes	232 days	62 years	IDC with lymphovascular invasion	Grade 2	ER^+^/PR^+^/Her2+
7	5/6	72 days	Yes	Yes	214 days	77 years	IDC	Grade 2	ER^-^/PR^-^/Her2^-^
8	4/6	35 days	Yes	Yes	248 days	63 years	IDC	Grade 2	ER^+^/PR^+^/Her2^-^
9	6/6	46 days	Yes	Yes	279 days	66 years	IDC	Grade 1	ER^+^/PR^+^/Her2

Tumor formation in NUDE mice following bilateral injections into the mammary fat pad of tumorspheres that were derived from the original patient biopsy. The latency or palpable tumor formation is indicated in number of days post-injection of tumorspheres into the mammary fat pad. 'Passage in mice' indicates that the primary tumor could be serially transplanted into mice to form subsequent primary tumors following *in vitro *formation of tumorspheres prior to injection into the mammary fat pad. Metastasis was determined by detection of human chromosome 17 by PCR using DNA isolated from mouse organs collected from mice injected with tumorspheres into the mammary fat pad. Metastatic latency is the average number of days between the injection of tumorspheres into the mammary fat pad and the detection of metastatic lesions in all organs analyzed by H+E staining

### Tumor formation and serial transplantation in NUDE mice

To establish the presence of tumor-initiating cells within the isolated tumorspheres, cells were injected into the mammary fat pad of female NUDE mice in combination with Matrigel and mice were monitored for tumor formation. Since previous *in vitro *experiments suggested dissociation of the tumorspheres caused decreased viability of the cells (data not shown), non-dissociated tumorspheres were injected into the mammary fat pad. Based on cell counts performed during previous *in vitro *experiments, an estimate of 50-100 cells comprised a tumorsphere of 100 μm in diameter. All of the primary tumor samples stained with H+E were histologically evaluated by a pathologist (K.M.). The tumor formation capabilities of tumorspheres isolated from samples 1-3 were conducted in NOD/SCID female mice (data not shown). Tumor formation for samples 1-3 was not observed 3 months post-injection and extension of the experiment was terminated because of a high incidence of thymic masses in the NOD/SCID mice that a previous study described as lymphoma development [28]. Consequently, subsequent experiments were performed with the mouse strain to Nu/Nu (NUDE). Injection of Matrigel alone into the mammary fat pad of NUDE mice did not result in tumor formation (Figure 1B, arrows). Injection of 50 tumorspheres (estimated total cells injected: 1-5 × 10^3 ^cells) isolated from samples 5-9 in combination with Matrigel into the mammary fat pad resulted in formation of small, palpable tumors within 3 months post-injection (Figure 1C-D. arrows) that were maintained until the end of the experiment (approx. 9-12 months post-injection). Tumorspheres isolated from sample 4 did not form tumors when injected into the mammary fat pad in combination with Matrigel (Table 1). Tumorspheres were re-isolated from the tumors formed in the mammary fat pad by employing the same serum-free, non-adherent culture conditions used to isolate tumorspheres from patient core biopsies. Serial transplantation was demonstrated by the injection of re-isolated tumorspheres into the mammary fat pad of NUDE mice in combination with Matrigel. Samples 5-9 were serially transplanted in this manner three times through NUDE mice. In summary, the *in vitro *selection for tumorspheres prepared from human breast core biopsies contained bTICs that formed small tumors when injected into the mammary fat pad of NUDE mice. These tumors were serially transplantable through NUDE mice upon re-isolation of tumorspheres *in vitro *and re-injection of tumorspheres into the mammary fat pad of mice.

### Characterization of tumors formed in the mammary fat pad upon injection of tumorspheres

**Figure 2:**
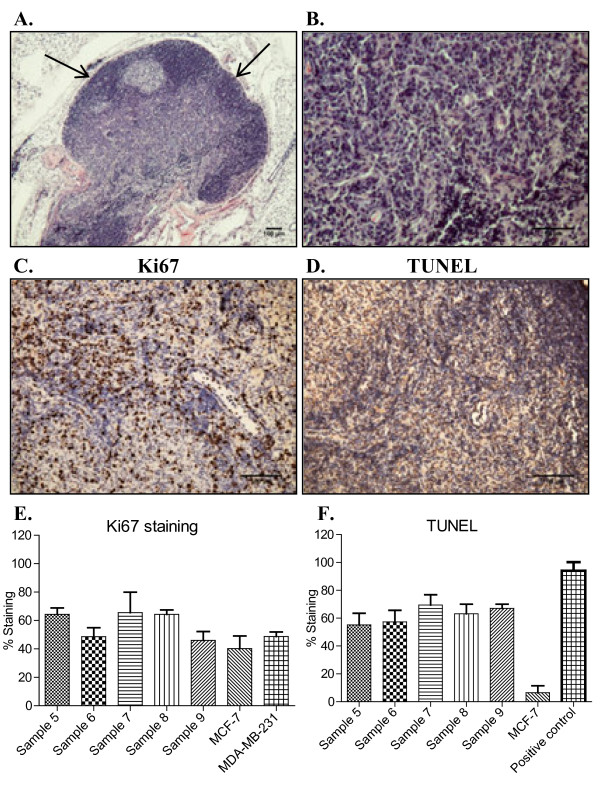
**Morphology, proliferation and apoptosis within primary tumors formed upon injection of tumorspheres**. **A, B**. H+E staining of 5 μm paraffin-embedded tumor sections derived from patient Sample 5 at 40× and 200×, respectively. **C, E**. IHC and quantitation for Ki67 were performed on 5 μm paraffin-embedded tumor sections derived from patients Samples 5-9. Ki67 positive cells and total number of cells were counted using Image J software in five randomly microphotographed images from each tumor sample and represented as the mean % positive cells with SD. **D, F**. TUNEL staining and quantitation (as described above) for apoptosis. MDA-MB-231 human breast cancer xenograft incubated with 4 U/ml DNase I was used as a positive control for TUNEL. Values are reported as mean +/- SD.

H+E staining was performed on sections of the primary tumors removed from the mammary fat pad. The edges of the tumors were often occupied by a dense population of cells as compared to areas closer to the center of the tumor that were less dense (Figure 2A, arrows). Tumors consisted of small tumor cells with pleomorphic nuclei that did not exhibit tubule formation (Figure 2A-B). IHC was performed on sections of the tumors to determine the number of proliferating cells using a rabbit monoclonal ki67 antibody. Over 40% of the cells were proliferating in sample 6 and 9, and over 60% of the cells were proliferating in samples 5, 7, and 8 (Figure 2C, E). To understand how a small tumor size could be maintained in the context of significant proliferation, terminal deoxynucleotidyl transferase (TdT)- mediated dUTP nick end labeling (TUNEL) was performed on sections of the tumors to determine the rate of apoptosis. The rates of apoptosis for samples 5-9 were similar to the rates of proliferation for each sample (Figure 2D, F) indicating a large degree of cell turnover in the tumors. In contrast, rapidly growing MCF-7 breast tumor xenografts did not display significant apoptosis (Figure 2F). A section from an MDA-MB-231 breast tumor xenograft incubated with 4 U/ml of DNase I at 37°C for 10 min was used as a positive control (Figure 2F, positive control).

**Figure 3:**
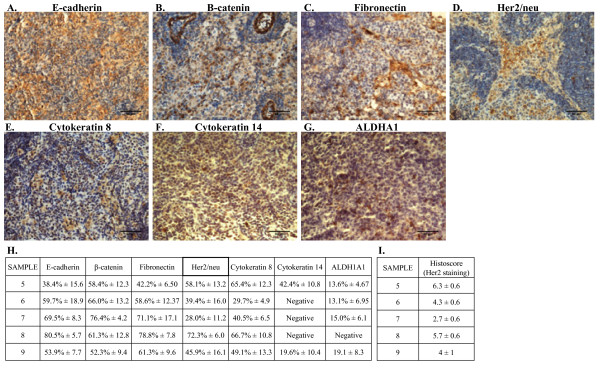
**Expression of markers for epithelial and mesenchymal lineages in tumor samples**. **A-G**. Representative IHC demonstrating patterns of expression of E-cadherin, fibronectin, Her2/neu, cytokeratin 8, cytokeratin 14, and aldehyde dehydrogenase 1 (ALDH1) in tumors formed upon the injection tumorspheres into the mammary fat pad of female NUDE mice. 200× magnification in all panels. **H**. Quantitation of positive staining and total number of cells were counted using Image J software in five randomly microphotographed images from each tumor sample and represented as the mean % positive cells with SD. **I**. Histological scoring of Her 2 expression within tumor samples. Values are reported as mean +/- SD.

The tumors were analyzed for the expression of a wide range or markers by IHC. Sections of MCF-7 and MDA-MB-231 tumor xenografts were used as controls for positive and negative staining by IHC for all antibodies (Additional file 2: Figure S2a-l). Expression levels for each marker were quantified using the MachBiophotonics ImageJ 1.42I program (as described in the Materials and Methods). All tumor samples were negative for estrogen receptor α (ERα) and the mesenchymal marker vimentin (data not shown). The cell adherens junction protein E-cadherin is normally expressed in the membrane of differentiated epithelial cells and more differentiated breast cancer cells. E-cadherin was observed at variable levels in the cytoplasm and nucleus in all tumor samples but not within the cell membrane (Figure 3A, H). β-catenin, a central mediator of the WNT pathway, binds to E-cadherin at the membrane in conjunction with a complex of proteins connecting the adherens junction to components of the cytoskeleton [29,30]. In the absence of membrane E-cadherin, β-catenin is either rapidly degraded or can translocate to the nucleus upon activation of WNT signaling. In corroboration with the aberrant cytoplasmic and nuclear E-cadherin staining, localization of β-catenin was also observed at variable levels in the cytoplasm and nucleus of all the tumor samples (Figure 3B, H). Although all the tumor samples were negative for vimentin, another mesenchymal marker fibronectin was detected in all samples (Figure 3C, H). Her2/neu was detected by IHC in all tumor samples however the extent of expression was highly variable between samples (Figure 3D, H). In addition to quantification using the MachBiophotonics ImageJ 1.42I program, Her2/neu expression within the tumor samples was expressed as a histoscore (as described in the Methods) (Figure 3I). Based on the reported histoscores, sample 7 exhibited the lowest expression of Her2 whereas sample 5 exhibited the highest expression of Her2 as compared to the other samples (Figure 3I). The detection of Her2/neu staining by IHC in the experimental tumors is not equivalent to the clinical diagnosis of Her2/neu positive tumors, which is based predominantly upon Her2/neu gene amplification. All tumors showed positive staining using an antibody to broad-spectrum cytokeratins at variable levels between tumors (data not shown) indicating the presence of epithelial lineage cells in the tumor. IHC for cytokeratin 8 and cytokeratin 14 was performed to determine the presence of luminal and myoepithelial cell lineages, respectively, within the tumors. Cytokeratin 8 was detected in all samples at variable levels (Figure 3E, H) whereas cytokeratin 14 was only detected in sample 5 and 9 (Figure 3F, H). These data indicate that tumors were comprised of mixed luminal and myoepithelial lineage tumor cells with some tumors negative for myoepithelial lineage tumor cells. Recently, aldehyde dehydrogenase (ALDH) has been implicated as a stem cell marker for both normal mammary cells and breast cancer cells [31,32]. IHC using a rabbit monoclonal antibody against ALDH1A1 demonstrated less than 20% of cells in all tumors expressed ALDH1A1 and no expression was detected in tumors formed from sample 8 (Figure 3G, H). These data, along with the cytokeratin staining, indicate that the tumors formed upon injection of tumorspheres into the mammary fat pad of NUDE mice did not entirely retain the primitive features of the tumorsphere [16,33], but instead exhibited marked heterogeneity in expression of lineage specific epithelial and mesenchymal markers. To confirm that tumors contained cells of human origin, IHC was performed using a mouse monoclonal antibody against human nuclear antigen (HNA) [34,35]. Sections of a human MDA-MB-231 breast tumor xenograft was used as a positive control for HNA staining (Additional file 3: Figure S3a); as a negative control, PBS used in place of the HNA primary antibody step for staining tumors formed upon injection of tumorspheres (Additional file 3: Figure S3b); sections of a mouse kidney incubated with the HNA antibody from a non-tumor bearing animal was also used as a negative control (Additional file 3: Figure S3c). In all tumors (samples 5-9), a majority of the cells stained positive for HNA as shown in the representative micrograph for sample 6 (Additional file 3: Figure S3d). Metastatic lesions within the liver and lung were also positive for HNA (Additional file 3: Figure S3e and f, respectively).

### Metastatic human cancer cells detected in mouse tissues

**Figure 4:**
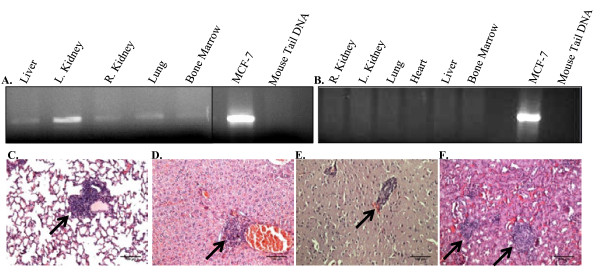
**Detection of micrometastasis of human cancer cells in mouse tissues**. **A**. PCR for a centromeric region in human chromosome 17 was used to detect human cells in mouse organs isolated from mice injected with tumorspheres into the mammary fat pad 3 months post-injection. Human DNA was detected in the lungs, kidneys, brain, bone marrow, and liver. DNA isolated from MCF-7 cells and mouse tail was used as a positive and negative control, respectively. **B**. PCR for DNA isolated from organs collected from a mouse injected with Matrigel alone was also used as a negative control. **C-F**. Micrographs representing micrometastasis in the lung, liver, brain and kidney, respectively, collected from mice previously injected with tumorspheres. 100× magnification in all panels. Arrows indicate micrometastases surrounded by normal mouse tissue.

Micrometastasis of human cancer cells to mouse kidney, liver, lung, brain and femurs (bone marrow) was assessed at 3 months post-injection using PCR to detect human chromosome 17 in the mouse tissues. Although no visual macrometastatic lesions were observed within any of the organs at 3 months post-injection, human DNA was detected in the kidneys, liver, lung, bone marrow (Figure 4A), and brain (data not shown). As a negative control, DNA was isolated from the organs of a mouse injected with Matrigel alone into the mammary fat pad; no signal was detected (Figure 4B). H+E staining demonstrated micrometastases within the lung, liver, brain and kidney (Figure 4C-F, respectively).

**Figure 5:**
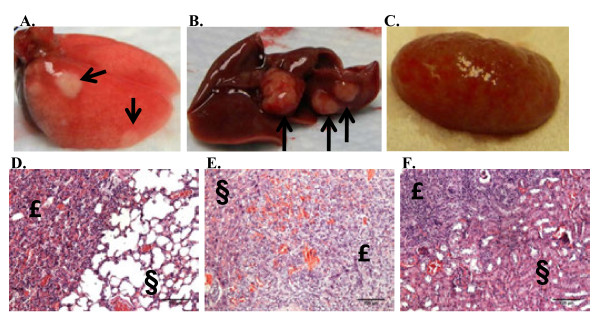
**Macrometastasis in the organs of mice injected with tumorspheres into the mammary fat pad**. **A-C**. Representative visual macrometastatic lesions detected in the lung, liver and kidney, respectively, 10 months post-injection of tumorspheres into the mammary fat pad of NUDE mice. **D-F**. H+E staining performed on 5 μm paraffin-embedded section of a lung, liver and kidney, respectively, illustrates macrometastatic lesions in the organs. Metastatic lesion indicated by £; normal mouse tissue indicated by §. 100× magnification in all panels.

Data from Figure 4 demonstrated that by 3 months post-injection of tumorspheres into the mammary fat pad, tumor cells had disseminated to distant organs. By 8 months post-injection, macrometastatic (visual) lesions were observed in the lung, liver and kidney for samples 5-9 (Figure 5A-C, respectively). H+E staining performed on sections of organs with visual metastatic lesions at the time of necropsy confirmed the presence of large metastatic lesions within the lung, liver and kidneys (Figure 5D-F, respectively).

### Organ tropism of the metastatic cells and the metastatic burden within the mouse organs

**Figure 6:**
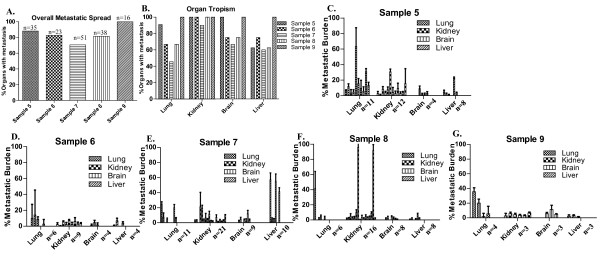
**Quantitation of the organ tropism of the metastatic cells and the metastatic burden within the mouse organs**. **A**. Comparison of the percentage of total organs that contained metastatic cells (Overall Metastatic Spread) for samples 5-9 as assessed by morphological analysis of H+E stained sections. The total number of mouse organs analyzed is indicated by n above each bar. **B**. Comparison of the percent of lungs, kidneys, brains and livers that contained metastatic cells (Organ Tropism) for samples 5-9 as assessed by morphological analysis of H+E stained sections. **C-E**. Graphical representation of the metastatic burden calculated for the total number of lungs, kidneys, brains and livers that were analyzed from mice injected with sample 5-9. The number of mouse organs analyzed is indicated by n in each graph. Each bar represents the calculated metastatic burden for one organ. The metastatic burden in each organ was calculated by dividing the pixels present in the metastatic lesion/s (x pixels) by the total pixels comprising the field of view (y pixels) then multiplying by 100 [(x pixels/y pixels)*100], resulting in a percent value. The percent metastatic burden was calculated from the means from 5 fields of view (100× magnification) per organ analyzed. Values are reported as mean +/- SD.

Paraffin-embedded sections of lungs, livers, kidneys and brains from samples 5-9 were stained with H+E to further determine the metastatic potential of the tumorspheres injected into the mammary fat pad. Since tumorspheres isolated from sample 1-3 did not form tumors in the mammary fat pad of NOD/SCID mice, the metastatic potential for these samples was not determined. A comprehensive analysis of metastasis was performed to: 1) determine the percentage of all organs examined (lung, kidney, brain, liver) that exhibited metastases for each of samples 5-9 (overall metastatic spread); 2) compare tropism of each sample to different organs, and; 3) quantitate the relative metastatic burden within each organ for each sample as a measure of the ability of metastatic cells to colonize organ sites and grow into larger lesions. The total number of organs with detectable metastases was counted to determine the overall metastatic spread for each sample. The data was further separated by the particular organ with detectable metastases (lung, liver, kidney and brain) to determine tropism. The metastatic burden within each organ was then quantified as described in the material and methods. The number (n) of lungs, kidneys, brains and livers analyzed for each sample is indicated in Figure 6C-G.

The percentage of all organs examined with metastasis (overall metastatic spread), without regard to the size of the metastatic lesions, was comparable between all samples (Figure 6A). Sample 7 demonstrated the lowest (71%), and sample 9 demonstrated the highest (100%) overall metastatic spread. Analysis of the percentage of organs with metastasis (without regard for the size of the metastatic lesions) revealed qualitative differences in the organ tropisms of the samples. Samples 5 and 9 exhibited the highest tropism to lung and brain as compared to the other samples and sample 9 also exhibited higher tropism to liver (100%) than any other sample (Figure 6B). Sample 5 additionally demonstrated the largest % metastatic burden in the lung compared to other tumor samples (compare lung % metastatic burden in Figure 6Cto Figures 6D-G). Although sample 9 demonstrated the highest tropism to liver (Figure 6B), the metastatic burden in the liver did not exceed 10% (Figure 6G) indicating that although sample 9 metastasized to liver in 100% of animals, the metastatic tumor cells did not develop into large metastatic lesions. In contrast, although sample 7 showed the lowest tropism to liver compared to other samples (60%, Figure 6B), the metastasized tumor cells yielded the greatest metastatic burden in the liver compared to other samples (Figure 6E). Sample 7 demonstrated the lowest tropism to the brain (Figure 6B) although the metastatic burden in the brain was comparable to the other samples (Figure 6E). Supplemental Figure 4 represents the % metastatic burden in each tissue for each sample as a function of the time the organs were removed after initial injection of tumorspheres into the mammary fat pad (Days post-injection). An increase in metastatic burden in any of the organs did not correlate with the number of days post-injection (Additional file 4: Figures S4a-e). However, sample 7 demonstrated larger metastatic lesions at earlier time points as compared to the other samples (Additional file 4: Figure S4c).

### Characterization of the metastatic cells within the mouse organs

**Figure 7:**
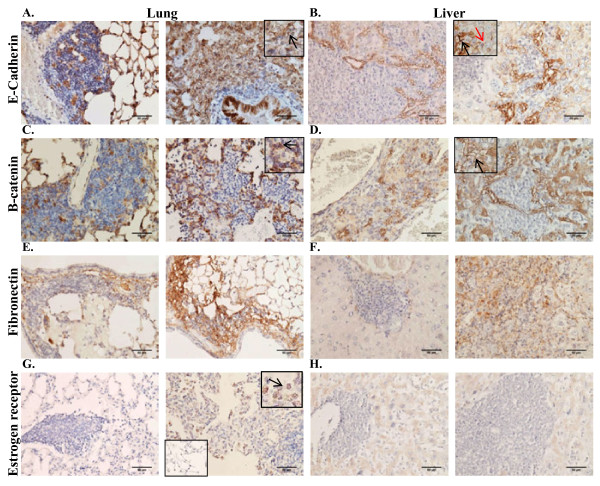
**Expression of markers for epithelial and mesenchymal lineages in metastatic lesions of lung and liver**. **A-B**. IHC performed on 5 μm paraffin-embedded sections of a lung and liver using a monoclonal anti-human antibody to E-cadherin. **C-D**. IHC performed on 5 μm paraffin-embedded sections of a lung and liver using a polyclonal anti-human antibody to β-catenin. **E-F**. IHC performed on 5 μm paraffin-embedded sections of a lung and liver using a polyclonal anti-human antibody to fibronectin. **G-H**. IHC performed on 5 μm paraffin-embedded sections of a lung and liver using a monoclonal anti-human antibody to ERα. **G**. Inset panel (bottom left) demonstrates negative staining in normal lung cells in proximity to the metastatic lesion in the lung. 200× magnification in all panels.

It is hypothesized that tumor cells acquire metastatic potential following an epithelial to mesenchymal transition (EMT) that permits local invasion and migration to distant metastatic sites. Once cells arrive at these metastatic sites, it is further hypothesized that tumor cells may undergo a reversion to reacquire epithelial characteristics that will permit survival and outgrowth at the ectopic site [30,36]. Therefore the expression of E-cadherin and β-catenin, two important modulators of EMT, was assessed by IHC within the metastatic lesions in the lung and liver. The localization of E-cadherin and β-catenin in the lung and liver was markedly different than the localization in the primary tumor (see Additional file 5: Table S1 for comprehensive comparison of marker expression profiles between primary tumor and metastatic lesions). In contrast to the predominantly cytoplasmic and nuclear localization of E-cadherin in the primary tumors, E-cadherin was detected in the membrane of the metastatic cells in the lung and liver although not all metastatic cells demonstrated E-cadherin staining (Figure 7A and 7B). In the liver, E-cadherin expression was most consistently observed in metastatic cells within close proximity to resident hepatocytes (Figure 7B, black arrow). Interestingly, hepatocytes within close proximity to metastatic cancer cells demonstrated stronger expression of E-cadherin in the membrane as compared to hepatocytes not proximal to metastatic cancer cells (Figure 7B, red arrow). Similar to localization of E-cadherin, β-catenin expression was also detected predominantly in the membrane of the metastatic cells in the lung and the liver (Figure 7C and 7D) in contrast to the cytoplasmic and nuclear localization in the primary tumor. The pattern and intensity of β-catenin staining was similar to that of E-cadherin within the same lung tissue samples (Figure 7C, black arrow). β-catenin was most strongly expressed in the membrane of metastatic cells within close proximity to hepatocytes in the liver, similar to the results with E-cadherin (Figure 7D, black arrow). Fibronectin, a component of the extracellular matrix (ECM), exhibited variable expression within metastatic lesions in the lung and the liver (Figure 7E and 7F). All metastatic lesions in the liver did not express ERα, consistent with the lack of ERα expression in the primary tumors (Figure 7H). However, metastatic lesions in the lung of mice injected with tumorspheres isolated from samples 6 and 8 demonstrated heterogeneous re-expression of ERα (Figure 7G, arrow).

## Discussion

Current research implies the presence of a population of cells in breast tumors with tumor-initiating capabilities. A number of cell surface markers have been used to sort for breast cancer cells with tumor-initiating capacity, including CD44 [6], CD133 [37], ALDH [31,38], CD90 [39,40], and CD117 (KIT) [41]. However, attempts to eliminate artifact may result in the biased selection of a small sub-population of cells present in disaggregated tissue. Presented in this study is an efficacious model for the *in vitro *isolation of tumorspheres, containing breast tumor-initiating cells, from human breast core biopsies. Injection of isolated tumorspheres into the mammary fat pad of NUDE mice resulted in formation and maintenance of small, palpable tumors that exhibited elevated proliferation and apoptosis. Within 8 months post-injection, widespread metastasis to mouse organs occurred most notably to liver, lung, and kidney. The cells within the tumors present in the mammary fat pad displayed heterogeneous expression of a range of markers for epithelial and mesenchymal differentiation that included cytokeratins, E-cadherin, β-catenin, fibronectin, as well as Her2/neu. Histological heterogeneity was observed both within individual tumors, and between tumors formed from tumorspheres isolated from different patient samples. Whereas primary tumors did not exhibit E-cadherin staining in cell membranes, a subset of tumor cells that had metastasized to the lung and the liver exhibited a re-distribution of E-cadherin to the membrane. These data describe a novel hetero-transplantation tumor model that exhibits a metastatic profile that recapitulates the development of metastasis in human breast cancer patients.

Tumorspheres were successfully isolated from all breast core biopsies including samples 1-3, which were not included in the data presented due to the lack of tumor formation and development of thymic lymphoma in NOD/SCID mice (as described in the Results section). Samples 5-9 tumorspheres were derived from patients with invasive ductal carcinoma (IDC), similar to other reports investigating the tumorigenic potential of breast cancer cells isolated as tumorspheres *in vitro *[16,33]. Although FACS has been used to isolate tumorigenic breast cancer cells from other breast cancer subtypes such as inflammatory breast cancer and lobular carcinoma [27], there are no reports demonstrating the successful isolation of tumor-initiating cells as tumorspheres from these breast cancer subtypes. When the host was changed to NUDE mice, the methods described herein reproducibly resulted in tumorsphere formation *in vitro*, and in tumor formation for all samples derived from primary breast core biopsies irrespective of ER/PR/Her2 status, tumor grade or stage. Tumorsphere number and size varied between samples, however differences could not be correlated with ER/PR/Her2 status, tumor stage or grade.

Tumorspheres isolated under select culture conditions likely contain a heterogeneous population of cells most of which will not likely have tumor-initiating capabilities. Therefore tumor formation upon the injection of a minimal number of cells that were not selected on the basis of epithelial origin or differentiation potential indicated the presence of tumor-initiating breast cancer cells within the tumorspheres. The injection of non-disaggregated tumorspheres, as opposed to single cell suspensions, establishes a novel method for the investigation of tumorigenicity *in vivo*. The absence of mechanical and/or enzymatic stress during preparation of the cells for injection improved the tumor cell viability. Therefore the injection of non-dissociated tumorspheres, in combination with the immune-deficient mouse model employed (NUDE mouse strain), contributed to the high tumor engraftment rates observed. However, future studies should determine differences in tumor formation and metastatic potential between single cell suspensions and non-disaggregated tumorspheres. Differences detected may reveal the effects of cell selection bias based on methodologies employed when preparing cells for injection.

The small primary tumor volume observed in the mammary fat pad suggested a low level of tumor cell proliferation. However the number of proliferating cells detected by Ki67 staining was high in all tumor samples. This high proliferation was offset by a number of cells undergoing apoptosis. This equilibrium between proliferation and apoptosis provided an explanation for the formation and long-term propagation of the small, palpable tumors, indicating the tumors were persisting in a state of tumor dormancy. However it also raised the question as to why the high level of proliferation observed in the tumors did not eventually overcome the apoptosis and result in larger tumor volumes with time. One possible explanation could be the inability of tumors to initiate neoangiogenesis that was manifest in high rates of apoptosis and proliferation that culminated with tumor dormancy [42,43]. Failure of the injected cells to efficiently recruit mouse stromal cells/endothelial cells would lead to nutrient and oxygen deprivation that would result in high levels of apoptosis to compensate for a high proliferation of the tumor cells. Alternatively, this disruption in homeostasis may occur as a later event during tumor progression, upon sufficient acquisition of somatic mutations within the differentiated progeny. Nonetheless, the metastatic phenotype demonstrated in this study implies that metastasis is an early event in tumorigenesis; conferring implications that challenge the linearity of breast tumor progression.

Traditional xenograft models derived from established breast cancer cell lines (e.g. MCF-7, MDA-MB-231) display fairly homogeneous organization, morphology, and protein expression patterns. In contrast, characterization of the tumors derived from primary tumorspheres presented here revealed heterogeneity among samples in morphology and marker expression. Because the tumorspheres exhibited stem-like properties, it might be expected that tumors in mice derived from the tumorspheres would exhibit similar morphology as the patient tumors. Because the tumorspheres exhibited stem-like properties, it might be expected that tumors in mice derived from the tumorspheres would exhibit similar morphology as the patient tumors. Although the human breast tumor samples ranged from grade 1-3, the tumors in the mammary fat pad did not exhibit all of the morphological characteristics of the patient tumors, such as tubule formation and ER and Her2 expression. This discrepancy is likely a result of the microenvironment within the mammary fat pad that is mostly devoid of the stromal and cellular components present in human mammary tissue, and instead is composed predominantly of adipose tissue. It is possible that the inclusion of human stromal/cellular components with the tumorspheres would more accurately recapitulate the microenvironment within human mammary tissue resulting in tumors that might exhibit tubule formation, ER/PR expression and other characteristics of low grade, more differentiated. In general, the edges of the tumors were lined with dense areas of cells that were also observed within the tumors as a dense ring of cells surrounding a more diffuse distribution of tumor cells (Figure 2). Although the expression patterns of most markers used in the characterization of the tumors did not correspond to this evident cellular organization, the expression of β-catenin did correlate with this organization displaying strong expression in the ring of cells encapsulating the diffuse population of cancer cells. All tumors in the mammary fat pad were negative for ERα despite the varied ERα status of the patient samples. During an EMT in breast cancer, E-cadherin expression in the membrane is reported to be lost or re-localized to the cytoplasm [29,44]. Consistent with a mesenchymal phenotype, in the primary tumors E-cadherin and β-catenin expression was localized in the cytoplasm and nucleus (Figure 3). Given that loss of E-cadherin expression in breast cancer is associated with an EMT and with the metastatic process, the unique expression pattern of E-cadherin in the present tumor model that exhibits a metastatic phenotype warrants further study.

Human metastatic cancer cells were detected by PCR at 3 months post-injection of tumorspheres into the mammary fat pad within the lung, liver, kidney, brain and femur (Figure 4). However the development of large metastatic lesions was observed by H+E staining in the lung, kidney and liver only. Comparison of the organ-specific percent metastasis to the metastatic burden within each organ revealed interesting results. Although sample 7 demonstrated the lowest percent metastasis overall and within the organs analyzed, the percent metastatic burden within each organ was comparable to or more often higher on average as compared to the other samples. In contrast, sample 9 demonstrated the highest percent metastasis overall and within the organs analyzed, however the percent metastatic burden within each organ was minimal compared to the other samples. These conflicting results elucidate a potential shortcoming in the current methods employed for predicting metastatic disease. The ability of cancer cells to metastasize from the primary site to distant sites may not accurately reflect the actual metastatic potential of the cells. Rather, understanding and predicting the adaptation of the cells that have arrived at the distant site of metastasis, may more accurately determine the potential for the development of overt metastatic disease. Samples 5 and 9, the only tumor samples derived from patient biopsies with clinical diagnosis of triple negative (ER^-^/PR^-^/Her2^-^), demonstrated the highest average metastatic burden within the organs. This observation may imply a growth advantage at distant sites for metastatic cells derived from triple negative tumors, although additional samples would be needed to demonstrate statistical significance. Despite the detection of metastasis by H+E staining in 60%-100% of brains analyzed, the metastatic burden was on average between only 1-3%. The blood-brain barrier (BBB) consists of tight junctions and adherens junctions between the brain endothelial cells, restricting the passage of substances from the bloodstream into the brain [45]. Impedance of entry into the brain by the BBB may contribute to the apparent extended dormancy period of the metastatic cells present in the brain, in combination with other influencing factors. Overall, the long duration from the time of detection of cancer cells in the organs (by PCR and/or histological staining) to the development of visual macrometastasis in mouse organs was similar to the delay in development of measurable metastasis in breast cancer patients following diagnosis of primary breast tumors [46,47]. The extended latency between the injection of tumorspheres and the development of macroscopic metastatic lesions is a limitation of this model. The injection of a higher number of cells may increase the time to tumor formation and the tumor volume in the mammary fat pad, however we speculate that the observed metastatic latency would not be affected. The organ microenvironment and intrinsic properties of the disseminated cells likely predominantly contribute to the observed metastatic latency. However secreted factors from the primary tumor within the mammary fat pad could also influence metastatic progression, therefore an increase in tumor volume may affect the proliferative state of disseminated tumor cells at distant sites. Future studies investigating the effects of larger tumor volumes on metastatic latency, possibly by the injection of increasing numbers of cells into the mammary fat pad, may elucidate the influence of secreted factors from the primary tumor on disseminated cancer cells. The detection of micrometastases in an array of organs with the development of macrometastases in only a select few of those organs suggests that mechanical/stochastic forces may permit entry of cancer cells into a range of organs; however metastatic cells will only survive and develop into overt lesions when present within a permissive, conditioned metastatic niche [20].

Whereas cytoplasmic and nuclear localization of E-cadherin and β-catenin was observed in the primary tumors, E-cadherin and β-catenin were re-expressed in the membrane in a subset of metastatic cells in the lung suggesting a possible reversion back to an epithelial phenotype (a mesenchymal to epithelial transition) [36,48]. E-cadherin and β-catenin expression was predominantly observed in the membrane of metastatic cells proximal to hepatocytes in the liver. Furthermore, hepatocytes proximal to metastatic cancer cells demonstrated stronger E-cadherin expression as compared to hepatocytes not within close proximity to metastatic cancer cells. Chao et al. demonstrated re-expression of E-cadherin in the membrane of MDA-MB-231 breast carcinoma cells *in vivo *and *in vitro *when in close proximity to hepatocytes [49]. Additionally, in prostate cancer models of metastasis to the liver, E-cadherin was shown to accumulate at the interface with hepatocytes [50,51]. These findings further support the importance of crosstalk between the cancer cells and native cells present within the organ. Fibronectin, a component of the extracellular matrix (ECM), plays an important role in cell migration, adhesion, maintenance of cell shape and wound healing. Fibronectin-dependent signaling has been linked to cancer cell dormancy, involved in the quiescent to proliferative switch [52,53]. Many of the smaller metastatic lesions in the liver did not demonstrate expression of fibronectin, however fibronectin was found variably expressed within the larger metastatic lesions. This observation could implicate fibronectin signaling as a possible mechanism involved in the development of macrometastatic lesions within the liver. The metastatic lesions within the liver were negative for ERα in all samples, consistent with the lack of expression within primary tumors. However in samples 6 and 8, metastatic lesions in the lungs of mice bearing primary tumors demonstrated variable re-expression of ERα, whereas the normal lung tissue did not exhibit expression of ERα. Interestingly, samples 6 and 8 were derived from patient biopsies clinically diagnosed as ERα positive. Previous clinical studies have reported differences in the expression of ERα between the primary patient tumor and the metastatic sites [54,55] although the underlying mechanisms to explain the altered expression are unknown. These results reinforce the importance of the microenvironment within the metastatic niche for influence on the differentiation state of the metastasized tumor cells and the expression of clinically relevant markers. The heterogeneity of the metastatic cells within the lung and the liver implied plasticity, whether innate or induced, during the metastatic process. Collectively, these findings demonstrate the complex integration of extrinsic and intrinsic factors affecting the behavior and phenotype of breast cancer cells during the metastatic process.

The present study data demonstrates a novel model for the study of human breast cancer breast cancer metastasis using samples obtained directly from patient biopsies. The metastatic phenotype demonstrated upon injection of tumorspheres into the mammary fat pad permits the study of all steps within the metastatic process. In particular, the development of macrometastatic lesions with organ-specific phenotypic distinctions provides a superior model for the investigation of organ-specific effects on metastatic cancer cell survival and growth. This model accurately recapitulates the metastatic latency observed clinically, permitting the development of therapeutics that target metastatic cells during dormancy prior to activation. Experimental manipulation of the organ-specific microenvironment could reveal molecular targets that are clinically accessible and biologically relevant. Furthermore, this model can be used to develop improved methods for the detection of micrometastatic cells and the prediction of metastatic disease.

## Conclusions

Primary breast tumor-initiating cells can be isolated as tumorspheres under non-adherent, serum free culture conditions from patient core biopsies independent of assigned grade or ER/PR/Her2 status. Isolated tumorspheres were tumorigenic in NUDE mice and had the capacity to metastasize from the primary site (i.e. mammary fat pad) to distant organs, such as the liver, lung, kidney, brain, and femur. Tumor cells at the metastatic sites exhibited organ-specific phenotypes that demonstrated plasticity of the metastatic cells dependent upon the organ microenvironment. This study describes a reproducible heterotransplant tumor model derived from patient biopsies that provides a novel method for the comprehensive study of breast cancer metastasis that better recapitulates the dormancy, complexity and heterogeneity within human breast cancer metastases.

## Abbreviations

BCSC: Breast cancer stem cell; ER: Estrogen receptor; bTIC: Breast tumor-initiating cell; HBSS: Hanks buffered saline solution; BSA: Bovine serum albumin; EGF: Epidermal growth factor; bFGF: Basic fibroblast growth factor; ESA: Epithelial specific antigen; PBS: Phosphate buffered saline; IHC: Immunohistochemistry; IDC: Invasive ductal carcinoma; H&E: Hematoxylin and eosin; TUNEL: Terminal deoxynucleotidyl transferase (TdT)- mediated dUTP nick end labeling; ALDH: Aldehyde dehydrogenase; HNA: Human nuclear antigen; EMT: Epithelial to mesenchymal transition; ECM: Extracellular matrix.

## Competing interests

The authors declare that they have no competing interests.

## Authors' contributions

CGM carried out the study design, animal necropsy, data collection, statistical analysis, data interpretation, manuscript preparation, and literature search. MJW provided the patient core biopsies and collected the consent forms from the patients. LC performed mouse injections. KM provided histological analysis of tissues. RP participated in study design and subsequent analysis. BGR organized the study as the director, manuscript preparation, and funding the collection. All authors read and approved the final manuscript.

## Funding

CGM was supported by a DOD Breast Cancer Research Program Predoctoral Traineeship Award BC093134. This project was supported, in part, by a seed grant from the Louisiana Cancer Research Consortium (BGR).

## Pre-publication history

The pre-publication history for this paper can be accessed here:

http://www.biomedcentral.com/1471-2407/12/10/prepub

## Supplementary Material

Additional file 1 **Figure S1. Characterization of cell surface marker expression of tumorspheres. A**. Immunocytochemistry (ICC) of tumorspheres prepared by formalin fixation and 5 μm paraffin-embedded sections using pre-conjugated antibodies against CD44-PE, and CD24-FITC. ICC for ESA-FITC was performed on tumorspheres prepared by centrifugation onto glass coverslips (cytospins). Tumorspheres demonstrate a CD44^+^/CD24^low-med^/ESA^+ ^cell surface marker phenotype. **B**. Isotype matched, pre-conjugated IgG control antibody mixture (IgG_1_-PE/IgG_2a_-FITC) was used as a negative control for ICC. 200× magnification in all panels. Click here for file

Additional file 2 **Figure S2. MCF-7 and MDA-MB-231 breast tumor xenografts used as controls for IHC**. **A-L **IHC performed on 5 μm paraffin-embedded sections of MCF-7 (**A-F**) and MDA-MB-231 (**G-L**) xenografts using rabbit monoclonal E-cadherin antibody (**A+G**), rabbit polyclonal β-catenin antibody (**B+H**), rabbit polyclonal fibronectin antibody (**C+I**), rabbit monoclonal Her2/ErbB2 antibody (**D+J**), rabbit polyclonal cytokeratin 8 antibody (**E+K**), and rabbit monoclonal cytokeratin 14 antibody (**F+L**). IHC results on MCF-7 and MDA-MB-231 xenograft sections were used as positive and negative controls for the IHC results on tumors formed after injection of tumorspheres in the mammary fat pad (Figure 3). All panels 200× magnification. Click here for file

Additional file 3 **Figure S3. Human nuclear antigen (HNA) staining detects human cells at in the primary tumor and at the metastatic sites**. **A**. 5 μm paraffin-embedded sections of MDA-MB-231 breast tumor xenograft used as a positive control for HNA (mouse anti-human nuclei monoclonal antibody) staining. **B**. Tumor sample matched negative control, with the replacement of the primary antibody with 1× PBS. **C**. Kidney isolated from a non-injected NUDE mouse, incubated with HNA to demonstrate human specificity with the lack of nuclear staining of the mouse kidney cells. **D**. Cells stain positive for HNA in 5 μm paraffin-embedded sections of a tumor removed from the mammary fat pad after injection of tumorspheres. **E-F**. HNA staining of 5 μm paraffin-embedded sections of metastatic lesions in the liver and lung, respectively confirms the human origin of the lesion, with the majority of nuclei staining positive. All panels 200× magnification. Click here for file

Additional file 4 **Figure S4. Correlation between metastatic burden and the time after injection of tumorspheres that mouse organs were removed**. Graphical representation of the percent metastatic burden, previously calculated as described in Figure 6, for each tissue for each sample as a function of the time the organs were removed after initial injection of tumorspheres into the mammary fat pad (Days post-injection). Values are reported as mean +/- SD. Click here for file

Additional file 5 **Table S1. Summary of heterogeneous marker expression between primary tumor (mammary fat pad) and metastatic lesions**. Tabular representation of the expression of E-cadherin, β-catenin, fibronectin, and ERα between samples in the primary tumor (mammary fat pad), metastatic lesions in the lung and the liver. Cytoplasmic localization is expressed as 'cyto' in the table. 'Variable' indicates the variability of staining within metastatic lesions. Click here for file
