# Supplementary figures and images for: "A novel in vivo model for the study of human breast cancer metastasis using primary breast tumor-initiating cells from patient biopsies"
Source: BMC Cancer. 2012 Jan 10;12:10. doi: 10.1186/1471-2407-12-10 (PMC3277457; doi:10.1186/1471-2407-12-10)

## Slide 1
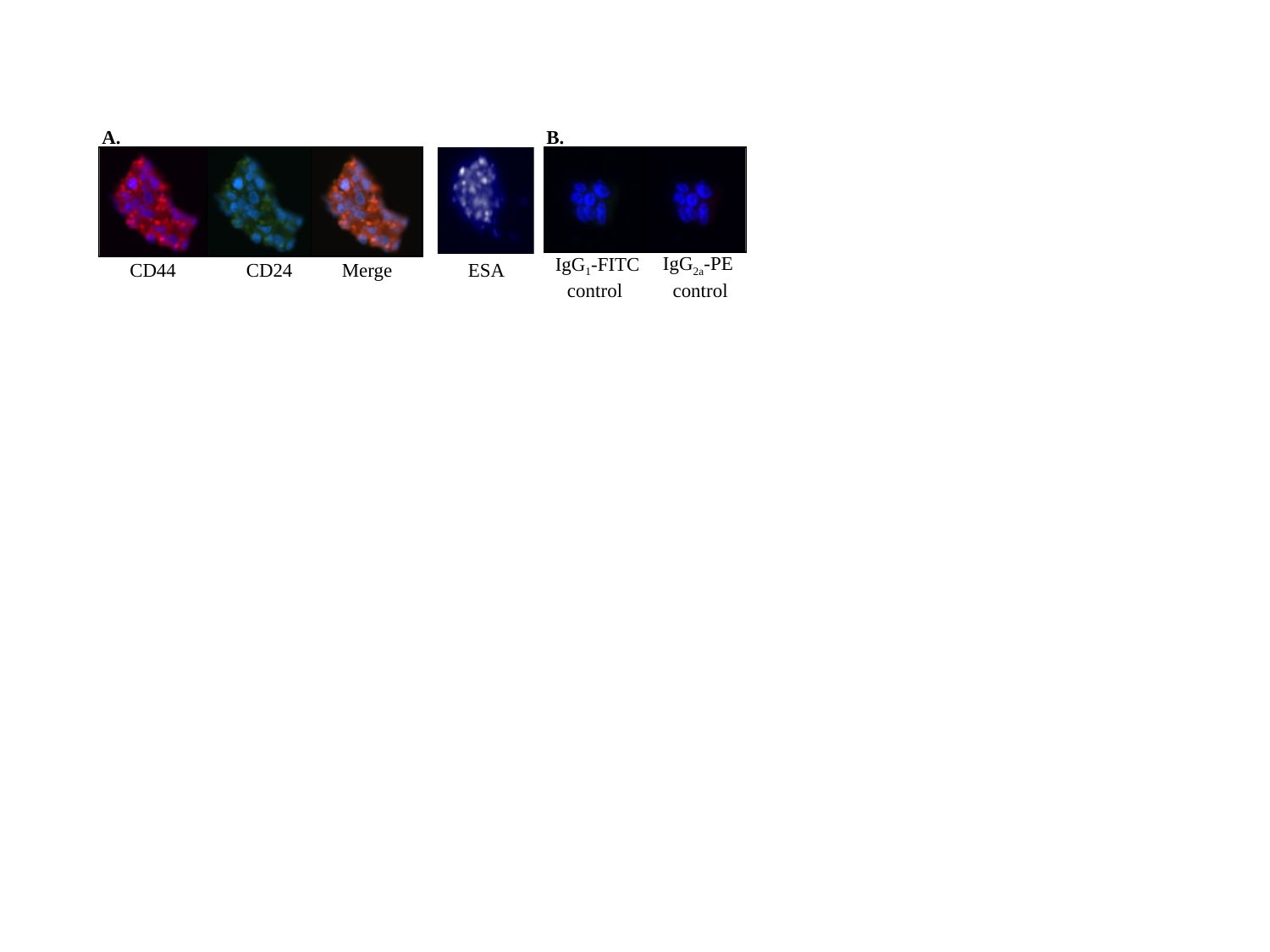

A.
B.
IgG1-FITC control
IgG2a-PE
 control
CD44
CD24
 Merge
ESA

Supplement: Additional file 1 — Figure S1. Characterization of cell surface marker expression of tumorspheres. A. Immunocytochemistry (ICC) of tumorspheres prepared by formalin fixation and 5 μm paraffin-embedded sections using pre-conjugated antibodies against CD44-PE, and CD24-FITC. ICC for ESA-FITC was performed on tumorspheres prepared by centrifugation onto glass coverslips (cytospins). Tumorspheres demonstrate a CD44+/CD24low-med/ESA+ cell surface marker phenotype. B. Isotype matched, pre-conjugated IgG control antibody mixture (IgG1-PE/IgG2a-FITC) was used as a negative control for ICC. 200× magnification in all panels. [file 1471-2407-12-10-S1.PPT]

## Slide 1
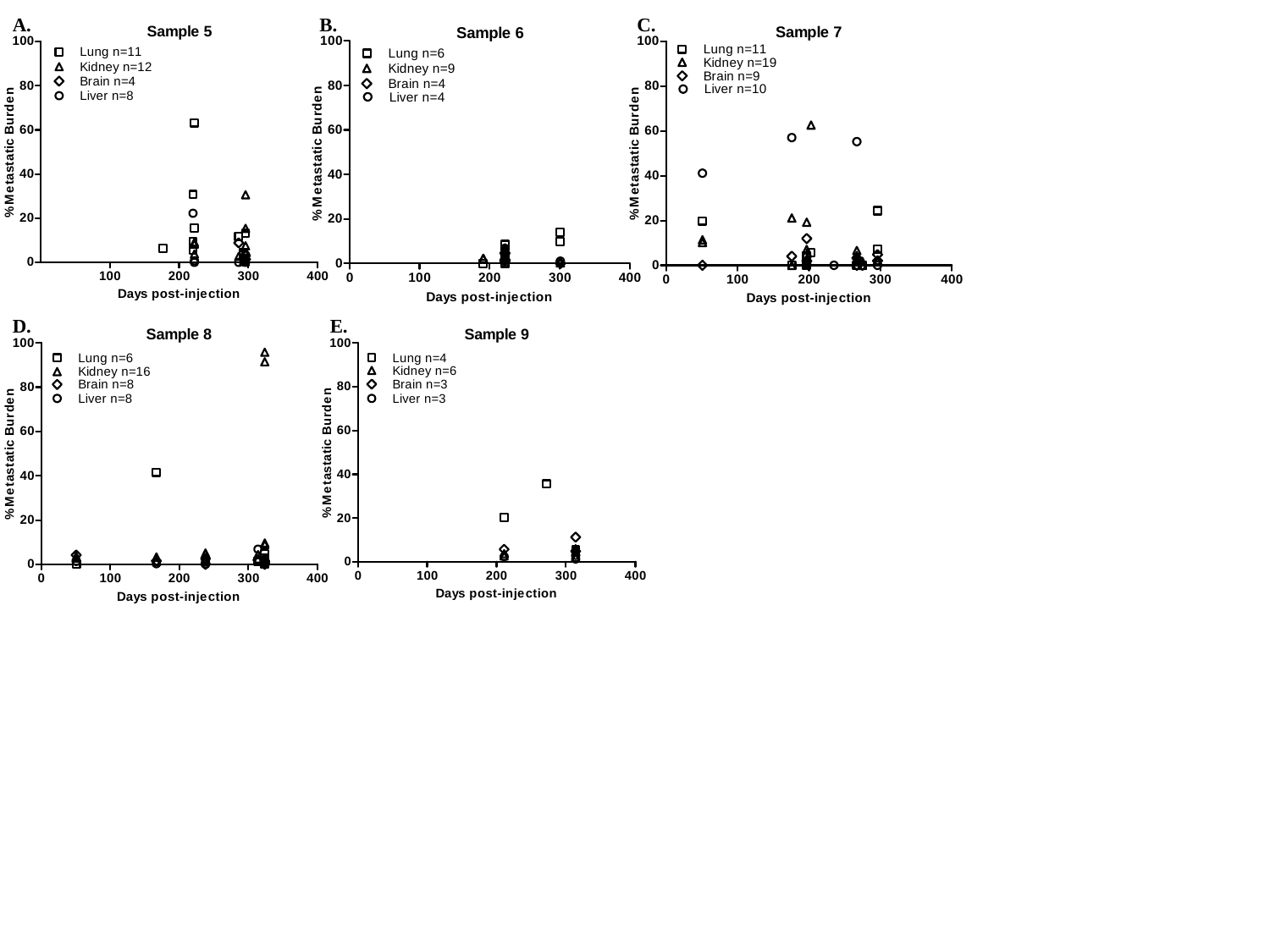

A.
B.
C.
D.
E.

Supplement: Additional file 4 — Figure S4. Correlation between metastatic burden and the time after injection of tumorspheres that mouse organs were removed. Graphical representation of the percent metastatic burden, previously calculated as described in Figure 6, for each tissue for each sample as a function of the time the organs were removed after initial injection of tumorspheres into the mammary fat pad (Days post-injection). Values are reported as mean +/- SD. [file 1471-2407-12-10-S4.PPT]
