# Supplementary material for: "A novel in vivo model for the study of human breast cancer metastasis using primary breast tumor-initiating cells from patient biopsies"
Source: BMC Cancer. 2012 Jan 10;12:10. doi: 10.1186/1471-2407-12-10 (PMC3277457; doi:10.1186/1471-2407-12-10)
Supplement: Additional file 2 — Figure S2. MCF-7 and MDA-MB-231 breast tumor xenografts used as controls for IHC. A-L IHC performed on 5 μm paraffin-embedded sections of MCF-7 (A-F) and MDA-MB-231 (G-L) xenografts using rabbit monoclonal E-cadherin antibody (A+G), rabbit polyclonal β-catenin antibody (B+H), rabbit polyclonal fibronectin antibody (C+I), rabbit monoclonal Her2/ErbB2 antibody (D+J), rabbit polyclonal cytokeratin 8 antibody (E+K), and rabbit monoclonal cytokeratin 14 antibody (F+L). IHC results on MCF-7 and MDA-MB-231 xenograft sections were used as positive and negative controls for the IHC results on tumors formed after injection of tumorspheres in the mammary fat pad (Figure 3). All panels 200× magnification. [file 1471-2407-12-10-S2.PPT]

## Slide 1
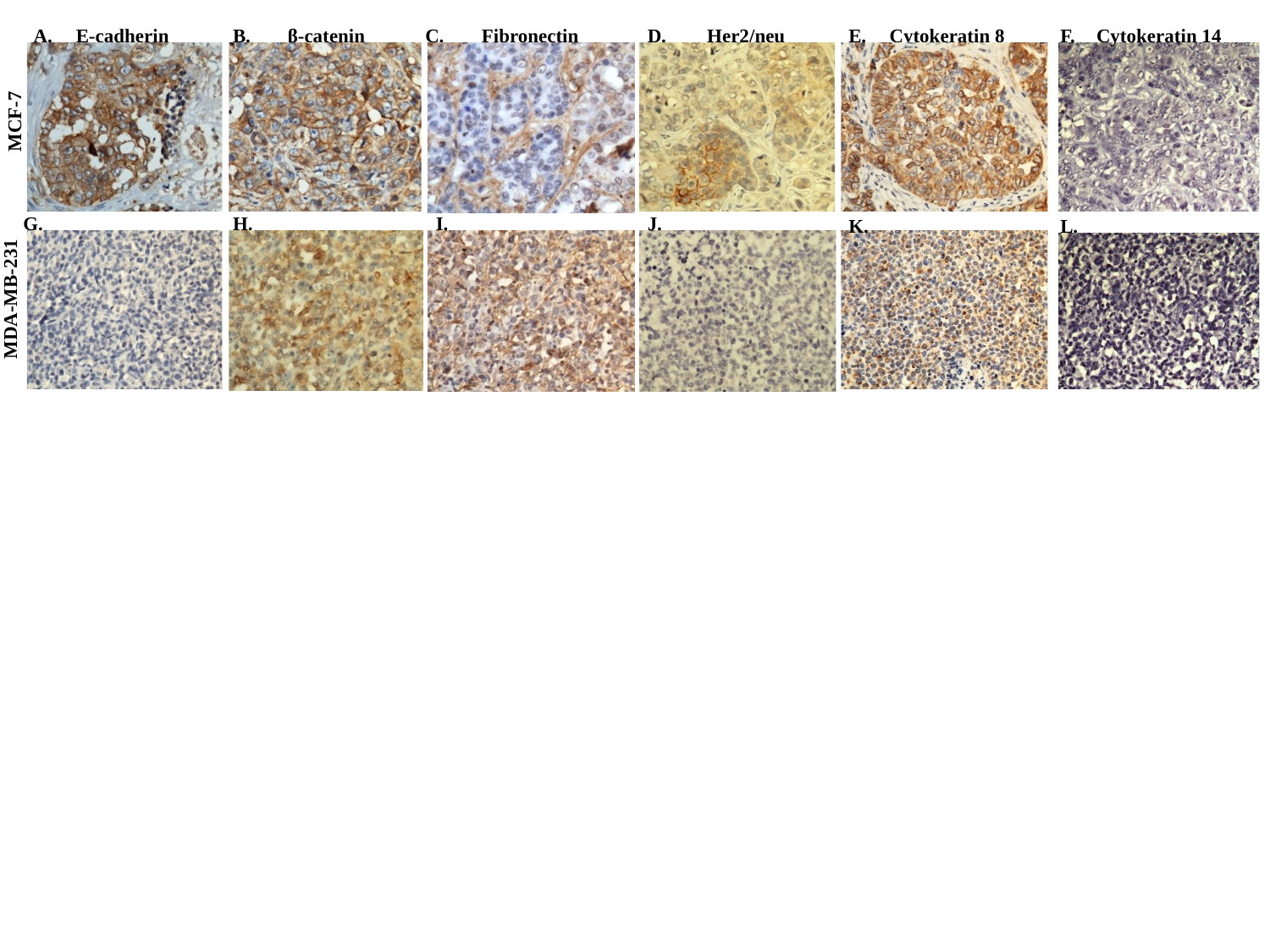

A.
E-cadherin
B.
β-catenin
C.
Fibronectin
D.
Her2/neu
E.
Cytokeratin 8
F.
Cytokeratin 14
MCF-7
G.
H.
I.
J.
K.
L.
MDA-MB-231
