# Supplementary material for: "A novel in vivo model for the study of human breast cancer metastasis using primary breast tumor-initiating cells from patient biopsies"
Source: BMC Cancer. 2012 Jan 10;12:10. doi: 10.1186/1471-2407-12-10 (PMC3277457; doi:10.1186/1471-2407-12-10)
Supplement: Additional file 3 — Figure S3. Human nuclear antigen (HNA) staining detects human cells at in the primary tumor and at the metastatic sites. A. 5 μm paraffin-embedded sections of MDA-MB-231 breast tumor xenograft used as a positive control for HNA (mouse anti-human nuclei monoclonal antibody) staining. B. Tumor sample matched negative control, with the replacement of the primary antibody with 1× PBS. C. Kidney isolated from a non-injected NUDE mouse, incubated with HNA to demonstrate human specificity with the lack of nuclear staining of the mouse kidney cells. D. Cells stain positive for HNA in 5 μm paraffin-embedded sections of a tumor removed from the mammary fat pad after injection of tumorspheres. E-F. HNA staining of 5 μm paraffin-embedded sections of metastatic lesions in the liver and lung, respectively confirms the human origin of the lesion, with the majority of nuclei staining positive. All panels 200× magnification. [file 1471-2407-12-10-S3.PPT]

## Slide 1
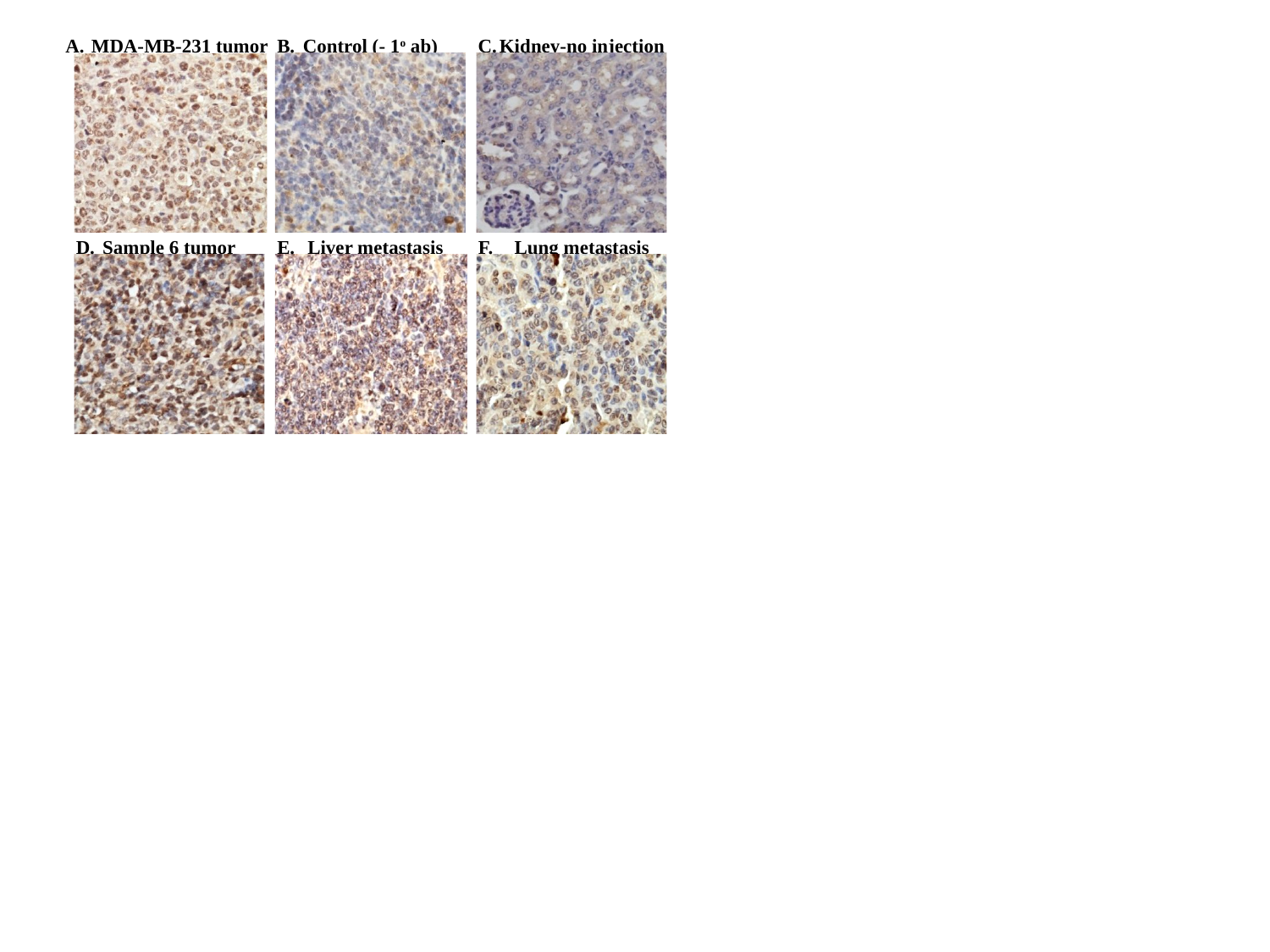

A.
MDA-MB-231 tumor
B.
Control (- 1o ab)
C.
Kidney-no injection
D.
Sample 6 tumor
E.
Liver metastasis
F.
Lung metastasis
