# Supplementary material for: "A novel in vivo model for the study of human breast cancer metastasis using primary breast tumor-initiating cells from patient biopsies"
Source: BMC Cancer. 2012 Jan 10;12:10. doi: 10.1186/1471-2407-12-10 (PMC3277457; doi:10.1186/1471-2407-12-10)
Supplement: Additional file 5 — Table S1. Summary of heterogeneous marker expression between primary tumor (mammary fat pad) and metastatic lesions. Tabular representation of the expression of E-cadherin, β-catenin, fibronectin, and ERα between samples in the primary tumor (mammary fat pad), metastatic lesions in the lung and the liver. Cytoplasmic localization is expressed as 'cyto' in the table. 'Variable' indicates the variability of staining within metastatic lesions. [file 1471-2407-12-10-S5.PPT]

## Slide 1
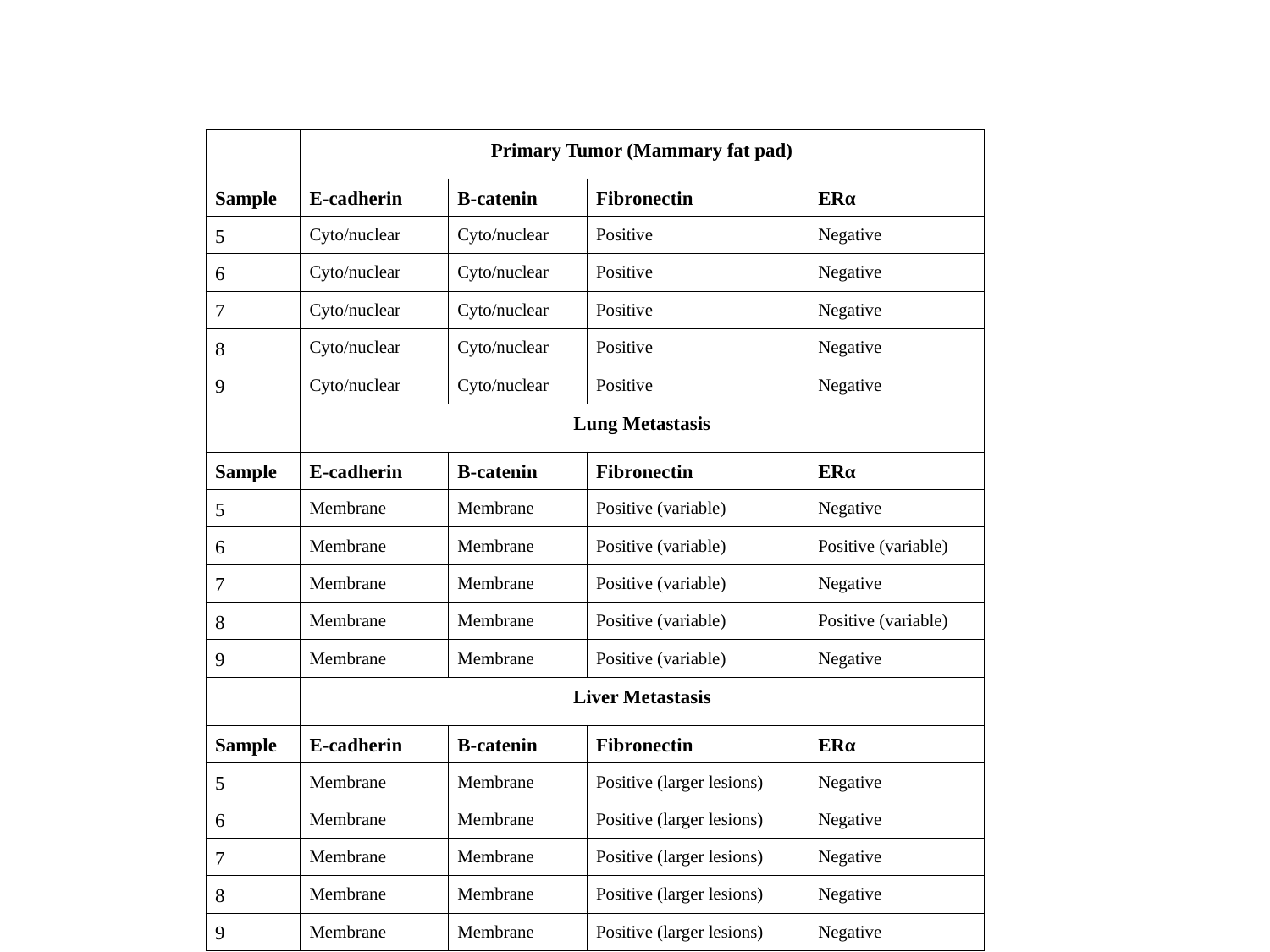

| | Primary Tumor (Mammary fat pad) | | | |
| --- | --- | --- | --- | --- |
| Sample | E-cadherin | Β-catenin | Fibronectin | ERα |
| 5 | Cyto/nuclear | Cyto/nuclear | Positive | Negative |
| 6 | Cyto/nuclear | Cyto/nuclear | Positive | Negative |
| 7 | Cyto/nuclear | Cyto/nuclear | Positive | Negative |
| 8 | Cyto/nuclear | Cyto/nuclear | Positive | Negative |
| 9 | Cyto/nuclear | Cyto/nuclear | Positive | Negative |
| | Lung Metastasis | | | |
| Sample | E-cadherin | Β-catenin | Fibronectin | ERα |
| 5 | Membrane | Membrane | Positive (variable) | Negative |
| 6 | Membrane | Membrane | Positive (variable) | Positive (variable) |
| 7 | Membrane | Membrane | Positive (variable) | Negative |
| 8 | Membrane | Membrane | Positive (variable) | Positive (variable) |
| 9 | Membrane | Membrane | Positive (variable) | Negative |
| | Liver Metastasis | | | |
| Sample | E-cadherin | Β-catenin | Fibronectin | ERα |
| 5 | Membrane | Membrane | Positive (larger lesions) | Negative |
| 6 | Membrane | Membrane | Positive (larger lesions) | Negative |
| 7 | Membrane | Membrane | Positive (larger lesions) | Negative |
| 8 | Membrane | Membrane | Positive (larger lesions) | Negative |
| 9 | Membrane | Membrane | Positive (larger lesions) | Negative |
